# Lipid keratopathy and septal abscess: Case report

**DOI:** 10.1097/MD.0000000000017802

**Published:** 2019-12-27

**Authors:** Sung Jae Heo, Jung Soo Kim, Sam Hyun Kwon, Jong Seung Kim

**Affiliations:** a Department of Otorhinolaryngology-Head and Neck Surgery, School of Medicine, Kyungpook National University, Kyungpook National University Chilgok Hospital; b Department of Otorhinolaryngology-Head and Neck Surgery, School of Medicine, Kyungpook National University, Kyungpook National University Hospital, Daegu; c Department of Otolaryngology-Head and Neck Surgery, College of Medicine, Chonbuk National University; d Research Institute of Clinical Medicine of Chonbuk National University-Biomedical Research Institute of Chonbuk National University Hospital, Jeonju, South Korea.

**Keywords:** epistaxis, lipid keratopathy, septal abscess, septal hematoma

## Abstract

**Rationale:**

Epistaxis is a common otorhinolaryngological emergency, but septal abscess has not been reported before as a complication of epistaxis.

**Patient concerns:**

We report a case of a 51-year-old man complaining of nasal obstruction and facial numbness for 3 weeks. He had a history of epistaxis, and had been treated with electrocauterization of the left nasal septum at a local clinic 1 month earlier.

**Diagnoses:**

On nasal endoscopy, swelling of the septum was noticed; computed tomography (CT) was performed, and revealed a septal abscess.

**Interventions:**

The patient was treated with incision and drainage under local anesthesia. A left vertical hemitransfixion incision was made and 4 mL of purulent material was drained. There was no quadrangular septal cartilage.

**Outcomes:**

On the 5th postoperative day, the patient complained of blurred vision in his right eye. Visual acuity of the left eye was 0.5, but acuity of the right eye was finger count at 50 cm. Examination of the right eye revealed a whitish fan-shaped corneal opacity on the medial side with neovascularization, diagnostic of lipid keratopathy.

**Conclusion:**

Electrocautery of epistaxis should be performed carefully during hemostasis, and there should be careful follow-up after the procedure to detect the occurrence of septal hematoma or septal abscess. These conditions should be treated as early as possible to avoid further serious complications. Since lipid keratopathy is difficult to treat once it occurs, care should be taken to avoid a septal abscess.

## 1 Introduction

Epistaxis is a common disease in the United States and accounts for one in every 200 people visiting an emergency room. It has a prevalence of about 60% of the general population.^[1]^ The costs of preventing such epistaxis are very high, and are more than $100,000 a year on a pediatric basis.^[2]^ Treatment of epistaxis includes ligation, embolization, nasal packing, and topical vasoconstriction.[^1^,^2^] Electrocautery is commonly used for anterior nasal bleeding, and it is known that narrow focus and short duration electrocautery is helpful for preventing complications such as septal perforation.^[1]^ Very rarely, cases of septal hematoma or abscess have been reported after electrocautery for epistaxis.

We report a case of lipid keratopathy after incision and drainage (I & D) as a treatment for septal abscess, which in turn had occurred after electrocautery for the treatment of anterior nasal bleeding.

This study was approved by the institutional review board of Chonbuk National University Hospital, Korea. Informed written consent was obtained from the patient for publication of this case report and accompanying images.

## 2 Case report

**Figure 1:**
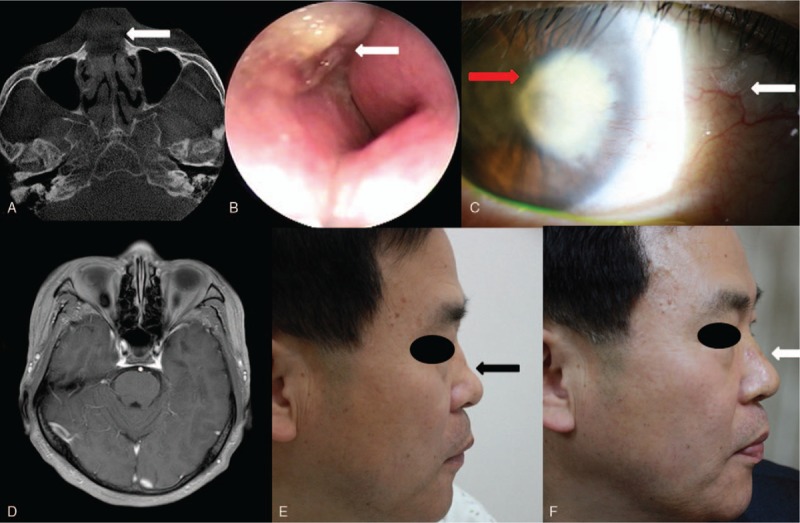
A. Computed tomography of the paranasal sinus. Enlarged septum with fluid content is indicated by a white arrow. Note the minimal air space in both nasal cavities. B. Nasal endoscopy of the left nasal cavity. Swelling of the septum can be seen (white arrow) despite previous incision and drainage. C. A slit-lamp examination revealed a fan-shaped whitish corneal opacity on the medial side of the right eye (red arrow). Neovascularization (white arrow) indicates a progressive lesion. D. Brain magnetic resonance imaging (MRI) showed a normal sigmoid sinus. E. A preoperative lateral photograph of the patient. Note the saddle nose caused by the septal abscess and septal necrosis (black arrow). F. A postoperative lateral photograph of the patient. Implanted costal cartilage now supports the saddle nose and necrotic septum (white arrow).

A 51-year-old man presented at our department having experienced nasal obstruction and facial numbness over the previous 3 weeks. He had a history of epistaxis, and had been treated with electrocauterization of the left nasal septum at a local clinic 1 month earlier. After that first treatment, he experienced headache and nasal obstruction for 2 weeks and visited another medical clinic. On examination, swelling of the septum was noticed and computed tomography (CT) was performed (Fig. 1A, white arrow). Cone beam CT revealed occupation of the nasal airway with fluid and severe swelling of the septum. The patient was treated with I & D under local anesthesia. In spite of the treatment with oral antibiotics, his symptoms did not improve, so he visited our department. On examination, there was diffuse swelling of the nasal dorsum and severe swelling of the septum (Fig. 1B, white arrow). The nasal cavity had no patency, so we undertook an emergency operation involving I & D under local anesthesia to treat the septal abscess.

He had no medical history or smoking history. On laboratory tests, white blood cell count was 8960/μL, erythrocyte sedimentation rate was 59 mm/hour, C reactive protein was 23.5 mg/L and serum cholesterol was 292 mg/dL. A left vertical hemitransfixion incision was made and 4 mL of purulent material was drained. There was no quadrangular septal cartilage. After saline irrigation, a silicon drain was inserted into the septal mucosa. Intravenous antibiotics were prescribed with 2 g of ceftriaxone every 12 hours and 0.5 g of metronidazole every 8 hours.

On the 5th postoperative day, the patient complained of blurred vision in his right eye. Visual acuity of the left eye was 0.5, but acuity of the right eye was finger count at 50 cm. Examination of the right eye revealed a whitish fan-shaped corneal opacity on the medial side with neovascularization, diagnostic of lipid keratopathy (Fig. 1C). Eye drops including moxifloxacin, prednisolone, and ketorolac were prescribed. We proposed that there was an infection or thrombus of the cavernous sinus; however, magnetic resonance imaging (MRI) showed no abnormal finding in the cavernous sinus or other vessels (Fig. 1D). An infectious organism or degenerative material from the nasal septal abscess may have been the source of lipid keratopathy via the ethmoid artery which originates from the ophthalmic artery. We postulated that regurgitation of material through the superior or inferior orbital fissure may have been the main cause of this serious condition.

The patient was concerned about the cosmetic appearance of his saddle nose caused by the septal abscess and septal necrosis (Fig. 1E), so reconstruction of the nasal dorsum and septum with 7th costal cartilage was performed after 8 weeks. The postoperative shape of his nose and septum was satisfactory (Fig. 1F), but the visual acuity and visual field defect in his right eye remained. Follow-up at 1 year was uneventful

## 3 Discussion

The most unusual aspect of this case report was that the problems started after bleeding control of epistaxis. In fact, when Kisselbach's plexus is the main cause of epistaxis, topical vasoconstrictor and direct compression are the first-line treatments. If epistaxis does not stop after initial treatment, non-absorbable nasal packing or electrocautery is applied to control bleeding.^[1]^ This patient had a rare case of septal abscess after electrocautery for epistaxis. If the patient had been monitored (or followed up) more closely in the local hospital after electrocautery, the occurrence of septal hematoma or septal abscess would have been prevented.

If a septal hematoma is left untreated, a septal abscess may result leading to severe functional and aesthetic complications.^[3]^ Saddle nose is an unsatisfactory cosmetic appearance caused by the septal abscess and septal necrosis; it can be resolved by surgery without difficulty. In our patient, external nose reconstruction using rib cartilage was performed about 1 month after the septal abscess had already developed. The reconstruction was relatively good, and the patient had no complaints about the external shape of his nose. However, lipid keratopathy occurred on the 5th day after I & D of the septal abscess. Our case has shown that severe complications can be induced by a septal hematoma or septal abscess.

The relationship between lipid keratopathy and septal abscess has not yet been established. Lipid keratopathy is a disease in which fat accumulates in the cornea. When the fat accumulates in the center of the cornea, visual acuity is disturbed.^[4]^ The pathophysiology of lipid keratopathy can be divided into primary and secondary keratopathy. Secondary lipid keratopathy is more common due to leakage of lipids from newly formed corneal vessels after inflammation.^[5]^ We note this inflammation in this pathophysiology. In this patient, the septal hematoma was left untreated for more than 1 month, causing a septal abscess. It is important to pay close attention to the blood supply to the nasal septum here. Kisselbach's plexus is composed of the anterior ethmoidal artery, sphenopalatine artery, greater palatine artery, and facial artery. Of these vessels, the only feeding vessel for the internal carotid artery (ICA) is the anterior ethmoidal artery.

The anterior ethmoidal artery is a branch of the ophthalmic artery, and the ophthalmic artery is a branch of the ICA.^[6]^ Since veins usually travel alongside the arteries, there is no guarantee that inflammation of the septal abscess has not affected the ophthalmic vessels.

Another branch of the ophthalmic artery is the anterior ciliary artery, which travels along an extraocular muscle to the front of the eye and supplies blood to the cornea.^[7]^ Ultimately, it is difficult to eliminate the possibility that the blood supply that caused the inflammation in which lipid keratopathy occurred was refluxing in the anterior ethmoidal vessel responsible for the blood supply to the septum.

Cavernous sinus thrombosis most commonly results from contiguous spread of infection from the sinuses or middle third of the face, or less commonly from a dental abscess or orbital cellulitis.^[8]^ *Staphylococcus aureus* is the most common infectious microbe, found in 50% to 60% of cases.^[8]^ Cavernous sinus thrombosis is a clinical diagnosis; however, MRI with contrast is the modality of choice to confirm its presence and to differentiate it from alternatives, for example orbital cellulitis, which may have a similar clinical presentation. Because of the suspicion of sequelae of septal abscess in this patient, evaluation of the cavernous sinus with MRI was performed, but no particular problem was found. This is probably because the inflammatory material only caused a problem in the ophthalmic vessel, and did not affect the cavernous sinus, since the patient was continuously on antibiotics during the admission period.

In this patient, septal hematoma developed 2 weeks after the first nasal trauma (electrocautery of septum), resulting in septal abscess 4 weeks after the first trauma, and eventually lipid keratopathy 5 weeks after trauma. Usually, secondary lipid keratopathy is usually associated with previous ocular disease or injury. According to the previous literature, there is no report that lipid keratopathy has been associated with nose, but it is reported that infection may be the cause.^[9]^ Goh et al reported a successful case of lipid keratopathy after herpes zoster ophthalmicus.^[9]^ It was developed during the 7 years healing process, which was successfully treated with photodynamic treatment. In our patient, corneal transplantation was recommended, but the patient refused, and the visual acuity and visual field defect in his right eye remained unchanged.

## 4 Conclusion

Electrocautery of epistaxis should be performed carefully during hemostasis, and there should be careful follow-up after the procedure to detect any occurrence of septal hematoma or septal abscess. These conditions should be treated as early as possible to avoid further serious complications. Since lipid keratopathy is difficult to treat once it occurs, care should be taken to avoid a septal abscess.

## Author contributions

**Conceptualization:** Jong Seung Kim, Sung Jae Heo, Jung Soo Kim.

**Data curation:** Jong Seung Kim, Sung Jae Heo, Jung Soo Kim.

**Formal analysis:** Jong Seung Kim, Sung Jae Heo, Jung Soo Kim.

**Funding acquisition:** Sung Jae Heo, Jung Soo Kim.

**Investigation:** Jong Seung Kim, Sung Jae Heo.

**Methodology:** Jong Seung Kim, Sung Jae Heo.

**Project administration:** Jong Seung Kim, Sung Jae Heo.

**Resources:** Sung Jae Heo, Sam Hyun Kwon.

**Software:** Jong Seung Kim, Sung Jae Heo, Sam Hyun Kwon.

**Supervision:** Jong Seung Kim, Sam Hyun Kwon.

**Validation:** Jong Seung Kim, Sam Hyun Kwon.

**Visualization:** Jong Seung Kim, Sam Hyun Kwon.

**Writing – original draft:** Jong Seung Kim, Sam Hyun Kwon.

**Writing – review & editing:** Jong Seung Kim.
